# Detection of circulating tumor HPV-DNA in patients with HPV-positive sinonasal and nasopharyngeal carcinoma

**DOI:** 10.2340/1651-226X.2026.46393

**Published:** 2026-10-20

**Authors:** Jakob Myllerup Jensen, Simone Kloch Bendtsen, Kathrine Kronberg Jakobsen, Katalin Kiss, Maria Rossing, Christian Grønhøj, Christian von Buchwald

**Affiliations:** a Department of Otorhinolaryngology, Head and Neck Surgery and Audiology, Copenhagen University Hospital – Rigshospitalet, Copenhagen, Denmark; b Department of Pathology, Copenhagen University Hospital – Rigshospitalet, Copenhagen, Denmark; c MDxCORE, Department of Clinical Biochemistry, Copenhagen University Hospital – Rigshospitalet, Copenhagen, Denmark; d Department of Clinical Medicine, Faculty of Health and Medical Sciences, University of Copenhagen, Copenhagen, Denmark

**Keywords:** HPV, ctHPV-DNA, biomarker, sinonasal carcinoma, nasopharyngeal carcinoma

## Abstract

**Background:**

Approximately 30% of sinonasal and nasopharyngeal carcinoma cases are HPV-associated, yet studies investigating circulating tumor HPV-DNA (ctHPV-DNA) among these patients are scarce. The aim of this study was to investigate the sensitivity of ctHPV-DNA in pretreatment blood samples from patients with HPV-positive (HPV+) sinonasal and nasopharyngeal carcinoma.

**Methods:**

Patients with a confirmed HPV+ sinonasal or nasopharyngeal carcinoma treated at the Department of Otorhinolaryngology, Head and Neck Surgery and Audiology at Rigshospitalet, Copenhagen, Denmark in the period 2017–2025 were included in this study. Blood samples were drawn in Streck tubes before treatment initiation and analysed for the presence of ctHPV-DNA by Droplet digital PCR (ddPCR).

**Results:**

Fifteen patients with primary HPV+ sinonasal or nasopharyngeal carcinoma were included. Except for two cases of HPV-related multiphenotypic carcinoma, all cases were squamous cell carcinoma. HPV16 was most prevalent and found in 6 patients (40%). HPV18 (*n* = 2), HPV33 (*n* = 1), HPV35 (*n* = 3), HPV45 (*n* = 1), and HPV56 (*n* = 2) were also represented. The sensitivity of detecting ctHPV-DNA in the pretreatment blood samples of the included patients was 80%. The level of ctHPV-DNA increased significantly with increasing T-category.

**Conclusion:**

ctHPV-DNA was detected in pretreatment blood samples in 80% of patients with HPV+ sinonasal and nasopharyngeal carcinoma. This supports further investigation of ctHPV-DNA as a biomarker for post-treatment surveillance in these patients.

## Introduction

Circulating tumor HPV-DNA (ctHPV-DNA) is gaining increasing clinical utility, particularly in HPV-positive (HPV+) oropharyngeal squamous cell carcinoma (OPSCC). However, reports on utility in HPV-associated sinonasal and nasopharyngeal carcinoma remain sparse. HPV+ tumors account for approximately 30% of sinonasal and nasopharyngeal carcinomas and their incidence is increasing, highlighting the potential of ctHPV-DNA in this patient group as well [1–4]. In this study, we investigate sensitivity of pretreatment detection of ctHPV-DNA among patients with HPV+ sinonasal and nasopharyngeal carcinoma.

## Material and methods

Patients with a confirmed primary HPV+ sinonasal or nasopharyngeal carcinoma treated at the Department of Otorhinolaryngology, Head and Neck Surgery and Audiology at Rigshospitalet, Copenhagen, Denmark in the period 2017–2025 were included in this study. HPV-status is not analysed routinely for sinonasal and nasopharyngeal carcinomas at Rigshospitalet. The included patients represent cases randomly selected for analysis.

Blood samples were drawn in Streck BCT CE DNA tubes from all patients prior to treatment initiation. A total of 2 × 10 mL blood was collected from each patient. Plasma was isolated and DNA extracted. Droplet digital PCR (ddPCR) was performed to assess the presence of DNA from the specific HPV-genotype that was found in the primary tumor of the patients, using a previously validated assay for HPV16, 18, 33, 35, and 45 [5, 6]. For HPV56 a custom primer/probe from BioRad (Assay ID: dCNS162679557) was used. Samples (x100 dilution) were considered positive according to the manufacturer’s definition if more than five droplets were positive. Samples that had fewer than five positive droplets or were only positive in undiluted form were defined as borderline positive.

Spearman’s rank correlation coefficient was used to assess the association between T-category and ctHPV-DNA levels, while the Jonckheere–Terpstra test was used to evaluate an ordered trend across T-categories.

## Results

Ten patients with HPV+ sinonasal carcinoma and five patients with HPV+ nasopharyngeal carcinoma were included at primary diagnosis. The median age at diagnosis was 56 years (range: 37–86 years), the majority were men (*n* = 10; 67%) and diagnosed in low TNM-categories (see Table 1). HPV16 was most prevalent and found in six patients (40%), followed by HPV35 (*n* = 3, 20%). The remaining cases were positive for HPV18, 33, 45, or 56. Thirteen cases were squamous cell carcinoma, the remaining two were HPV-related multiphenotypic carcinoma.

**Table 1:** Baseline characteristics of HPV+ sinonasal and nasopharyngeal carcinoma patients treated at the Department of Otorhinolaryngology, Head and Neck Surgery and Audiology at Rigshospitalet, Copenhagen, Denmark in the period 2017–2025.

Characteristic	Overall *N* = 15^a^	Nasopharynx *N* = 5^a^	Sinonasal *N* = 10^a^
**Sex**			
Female	5	1	4
Male	10	4	6
**Age**	56 (37–86)	56 (51–62)	56 (37–86)
**HPV**			
16	6	2	4
18	2	1	1
33	1	0	1
35	3	2	1
45	1	0	1
56	2	0	2
**Histopathology**			
HPV-related multiphenotypic carcinoma	2	0	2
Squamous Cell Carcinoma	13	5	8
**T**			
1	8	1	7
2	4	2	2
3	2	2	0
4	1	0	1
**N**			
0	12	2	10
1	2	2	0
2	1	1	0
**M**			
0	15	5	10

a *n*; Median (Min – Max); T: T-category; N: N-category; M: M-category.

In 12 patients, the pretreatment blood sample was either positive or borderline positive for the presence of ctHPV-DNA, yielding a sensitivity of 80%. The ctHPV-DNA level varied substantially among patients with positive samples, ranging from 0.2 to 2103861.5 copies/mL, with a median of 2.0 copies/mL. Spearman’s rank correlation revealed a significant positive association between T-category and ctHPV-DNA levels (*ρ* = 0.619, *p* = 0.032). This finding was supported by a Jonckheere–Terpstra test, which demonstrated a significant increasing trend in ctHPV-DNA levels with increasing T-category (JT = 37, *p* = 0.022).

The three cases that had negative pretreatment blood samples all had sinonasal squamous cell carcinoma diagnosed in T-category 1 or 2, and N-category 0. HPV-subtypes found in their primary tumor were HPV16, HPV45, and HPV56.

## Discussion

In this study we found a sensitivity of 80% in detecting ctHPV-DNA in pretreatment blood samples from 15 HPV+ sinonasal and nasopharyngeal carcinoma patients. Aside from a few case reports on the topic, only one prior study from 2023 has reported sensitivity of ctHPV-DNA in this patient group [7–9]. This study by Naegele et al. included nine patients and found a 100% sensitivity for pretreatment detection. The same study also monitored six patients after end of treatment, of whom two had treatment failure, which was correctly predicted by ctHPV-DNA. Our study reports a lower sensitivity, also lower than most recent reports in HPV+ OPSCC [6, 10–12]. While the relation between HPV and OPSCC is very well established, the relation with sinonasal and nasopharyngeal carcinoma is less clear. An explanation of the lower sensitivity could be that HPV in some cases is present without playing an active role in tumorigenesis. However, there are studies showing transcriptionally active HPV in sinonasal carcinoma, and a possibly better prognosis of HPV+ sinonasal carcinoma compared to HPV-negative cases, similar to that of HPV+ OPSCC [2, 13, 14]. The lower sensitivity could also be explained by lower TNM-categories of the patients included in this study compared to the study by Naegele et al. In this study 80% were diagnosed in T-category 1 or 2, compared to 60% of patients diagnosed in T-category 3 or 4 in the study by Naegele et al. This is also the case for nodal involvement where 80% of the patients were diagnosed in N-category 0, while half of the patients in the study by Naegele et al. had nodal involvement at diagnosis. Hence, we suspect that higher tumor load leads to increased sensitivity of detection of ctHPV-DNA. This is also supported by the significant increase in ctHPV-DNA levels with increasing T-category found in this study.

Despite the lower sensitivity, we believe that an 80% detection rate still supports the potential clinical utility of ctHPV-DNA in the follow-up of patients with HPV+ sinonasal and nasopharyngeal carcinoma. Besides the aforementioned studies, there are currently no reports on sensitivity of detecting recurrence of HPV+ sinonasal or nasopharyngeal carcinoma. Due to its rarity, studies on this could benefit from a multicenter or multinational setting.

In conclusion, among 15 patients with HPV+ sinonasal and nasopharyngeal carcinomas, ctHPV-DNA was detected in pretreatment blood samples in 80% of cases. Although this detection rate is lower than previously reported, it indicates that ctHPV-DNA is detectable in the majority of these patients, warranting further investigation of ctHPV-DNA for treatment-response evaluation and early detection of recurrence.

## Data Availability

According to Danish data protection law, data from this study are not allowed to be made publicly available. Transfer of data requires permission from the Danish data protection authorities for the specific transfer. This can be applied for upon request to the corresponding author.
